# Artificial Intelligence-Based Assistance System for Visual Inspection of X-ray Scatter Grids

**DOI:** 10.3390/s22030811

**Published:** 2022-01-21

**Authors:** Andreas Selmaier, David Kunz, Dominik Kisskalt, Mohamed Benaziz, Jens Fürst, Jörg Franke

**Affiliations:** 1 Institute for Factory Automation and Production Systems, Friedrich-Alexander University, 91058 Erlangen, Germany; david.kunz@faps.fau.de (D.K.); dominik.kisskalt@faps.fau.de (D.K.); joerg.franke@faps.fau.de (J.F.); 2 Technology Center for Power and Vaccuum Components, Siemens Healthineers, 91052 Erlangen, Germany; mohamed.benaziz@siemens-healthineers.com (M.B.); jens.fuerst@siemens-healthineers.com (J.F.)

**Keywords:** object detection, visual inspection, assistance system, industrial artificial intelligence application, X-ray scatter grid

## Abstract

Convolutional neural network (CNN)-based approaches have recently led to major performance steps in visual recognition tasks. However, only a few industrial applications are described in the literature. In this paper, an object detection application for visual quality evaluation of X-ray scatter grids is described and evaluated. To detect the small defects on the 4K input images, a sliding window approach is chosen. A special characteristic of the selected approach is the aggregation of overlapping prediction results by applying a 2D scalar field. The final system is able to detect 90% of the relevant defects, taking a precision score of 25% into account. A practical examination of the effectiveness elaborates the potential of the approach, improving the detection results of the inspection process by over 13%.

## 1. Introduction

In order to assess product quality in medical devices, numerous quality measures are carried out [1]. Vision-based controls are commonly applied, due to their nondestructive and noninvasive nature. In many small- and medium-series productions, these controls are still performed manually due to the high flexibility and high visual capabilities of human operators. However, the manual process has some weaknesses as human operators are prone to subjectivity, fatigue, and variability in daily performance.

Artificial intelligence (AI), in particular deep learning by convolutional neural network (CNN) architectures, creates the opportunity of automating or even improving the classification performance of existing visual inspection protocols. Compared to classical computer vision solutions, deep learning approaches have a number of advantages, such as the following:

- Potentially increased flexibility and capabilities of the model [2];
- Reduction in laborious manual tasks such as handcrafting image features [3];
- Generalization of development using existing model architectures or pretrained models [2,3];
- Adaption to changing conditions using updated dataset and retraining [4].

However, deep learning has some limitations and requires some preconditions:

- Limited explainability of the model predictions due to usage of black-box models [5];
- Constant conditions as covered by training dataset [6];
- In the case of supervised ML approaches, high quality of the ground truth data [7].

In order to utilize their advantages while considering their limitations, AI algorithms are frequently embedded within assistance systems. There, the capabilities can be used to their full potential, improving reliability, reproducibility, and process ergonomics but leaving final decisions to the tester. Thus, the AI is not fully autonomously executing any critical processes. Similar concepts are already known in the diagnosis processes of medical images [8]. In this paper, the results of an AI-based object detection application for the inspection process of X-ray scatter grids are presented.

**Figure 1:**
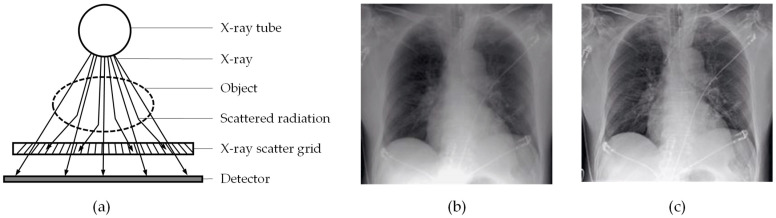
(**a**) Schematic view of X-ray image capturing process; based on [10] (**b**) X-ray image captured without scatter grid; (**c**) X-ray image captured with scatter grid.

X-ray scatter grids, also referred to as Potter–Bucky grids, are used in the X-ray image capturing process to filter out the scattered secondary radiation, letting only the nonscattered primary X-rays pass to the detector (see Figure 1a) [9]. This leads to enhanced image quality by increasing contrast and sharpness, as shown in Figure 1b,c contrasting X-ray images captured with and without applied scatter grid. Scatter grids are composed of alternating stripes of X-ray absorbent and transparent materials, which are slanted (as illustrated in Figure 1a) so that their angle corresponds to that of the primary radiation of the point source.

The paper is structured as follows: Section 2 presents various existing CNN-based object recognition applications and related work. In Section 3, use cases, system methodology, dataset properties, training strategy, and model optimization are outlined. Afterward, the system evaluation is detailed in Section 4. Finally, in Section 5, the results are summarized and an outlook on future work is given.

## 2. CNN-Based Object Detection

Object detection refers to the process of locating objects with a bounding box in an image. In this section, several fields of applications and algorithms used are presented.

### 2.1. Fields of Application

Object detection is used in a variety of applications, e.g., to automatically locate license plates in real time [11]; detect vehicles, pedestrians, traffic lights, and other objects for self-driving cars, as reviewed in [12]; or track objects such as balls for sport broadcasts [13]. In the context of X-ray image analysis, a variety of successful applications can be found in the fields of medical diagnosis, e.g., [14] uses a CNN-based approach for chest pathology identification, and security applications, which are collected and categorized in a survey by Akcay and Breckon in [15].

Object detection is also applied in manufacturing in fields such as quality management, e.g., the identification of missing and misaligned parts in an assembly in [16]; sorting, e.g., detecting objects for sorting on a conveyor belt in real-time in [17]; and crack detection, e.g., the inspection of pipes in nuclear power plants using remotely recorded videos [18].

### 2.2. Influential Algorithms and Related Work

A large driver for the application of object detection algorithms is the advances of CNN-based algorithms. Since the success of AlexNet [19] in 2012, algorithms such as YOLO [20] allow for near real-time assessment of images. Inception [21] introduced the application of multiple kernels with different sizes in parallel instead of linear convolutional layers increasing learning and abstraction capabilities. The Xception network [22] expands on this idea and decouples the traditional convolution layers into two pointwise and depthwise convolutions further improving accuracy and speed.

These and similar algorithms have also been integrated in several applications in the manufacturing context such as the following selected publications this paper is based on:

In [23] a deep neural network is applied to optical quality inspection of printing cylinders. The high-quality images are separated into 100 × 100 pixel-sized patches which are classified as OK or not-OK to identify the predominantly small defects. The developed approach reaches an accuracy rate of over 98%.

Defect detection for a benchmark dataset containing six image classes is performed in [24]. In the first step, an algorithm determines the image class. Then, the image is separated into patches with 50% overlap. Each patch is predicted as nondefective or defective by a separate algorithm for each class. If one of the patches is predicted as defective, the entire image is classified as defective.

In [18] a deep learning framework based on CNN and naïve Bayes data fusion is applied for weld crack detection using videos. Overlapping frames are predicted and fused based on spatiotemporal information, creating tuples of aggregated predictions from multiple video frames.

A similar sliding window approach is applied in [25] with the goal of reducing the need for labeled images and giving a defect segmentation as visual feedback in the quality inspection of solar panels. The images are separated into patches with 80% overlap and separately classified as “defective” and “non-defective”. For each patch, probability values greater than 0.6 are added to a new image with the same size as the original. A threshold is applied to this heatmap image to classify the entire image as defective or non-defective. Only the images that are considered as defective are re-evaluated by a human operator with highlighted regions of interest (ROIs). This approach improves the recall and precision from 42% and 44% at the patch level to 92% and 85% for the entire images.

## 3. Materials and Methods

**Figure 2:**
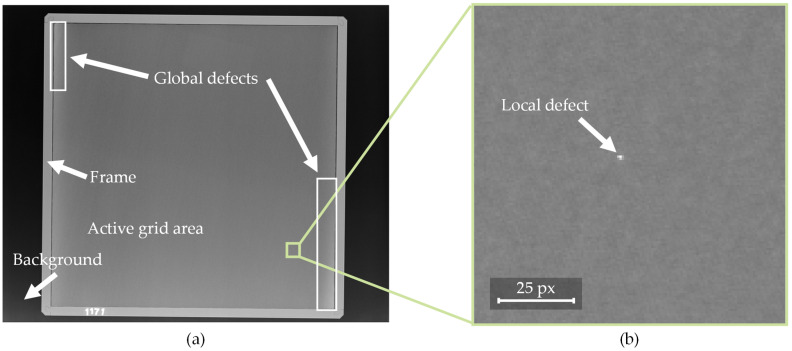
(**a**) Entire X-ray image including annotations for background, frame, active grid area, and two global defects; (**b**) subsection of (**a**) containing a highlighted local defect.

The goal of this work is to evaluate the capacity and suitability of CNN-based systems for detecting and localizing defects in the X-ray images of scatter grids. Therefore the implemented approach was developed and tested upon a real-world industrial production dataset. X-ray scatter grids are meant to enhance image quality (see Figure 1). However, defects or irregularities of the grid may result in artifacts on the X-ray images. To ensure grid quality, the grids are subjected to a visual inspection. This inspection is not carried out directly by inspecting the grid, but by inspecting a test image, which is taken with a very low X-ray dosage in order to amplify potential defects (see Figure 2). In a manual process, the operator inspects the test images using reference images of different defect types, which are illustrated in Figure 2.

**Figure 3:**
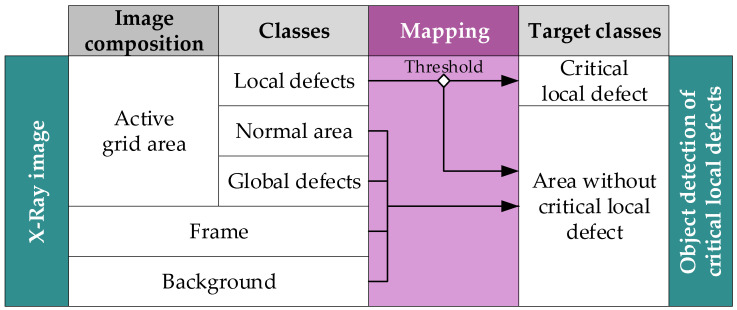
Composition of X-ray image and mapping to the target classes of the object detection.

Figure 2 shows a low dosage test image of a square scatter grid showing the active grid area as well as the grid’s frame. In general, two categories of defects can be distinguished. The first one is global defects, which extend over large parts of the image and are therefore clearly visible to the trained human eye. The second category is local defects, which are relatively small (2–8 pixels in diameter) compared to the test image size (4248 × 3480 pixels) and are therefore difficult and tedious to detect by the human eye. Therefore, the goal of the assistance system is to support this process by detecting local defects. Since the local defects vary in appearance, an ML approach is chosen over traditional methods, due to the ability of automated feature learning. Figure 3 shows the mapping of the described image components onto the target classes of the ML algorithm.

The X-ray image consists of five classes identified and defined based on a visual analysis in collaboration with process experts. Four of the five classes (normal areas, global defects, frame, and background) are considered as areas without local defects. Local defects vary in severity, based on which they are distinguished as critical or noncritical.

### 3.1. Methodology

The system itself was implemented in Python. For deep learning functionalities, the python API for Keras [26] combined with TensorFlow [27] as backend engine was utilized. Further machine learning functionalities of scikit-learn [28] and morphological operations of openCV [29] were used.

**Figure 4:**
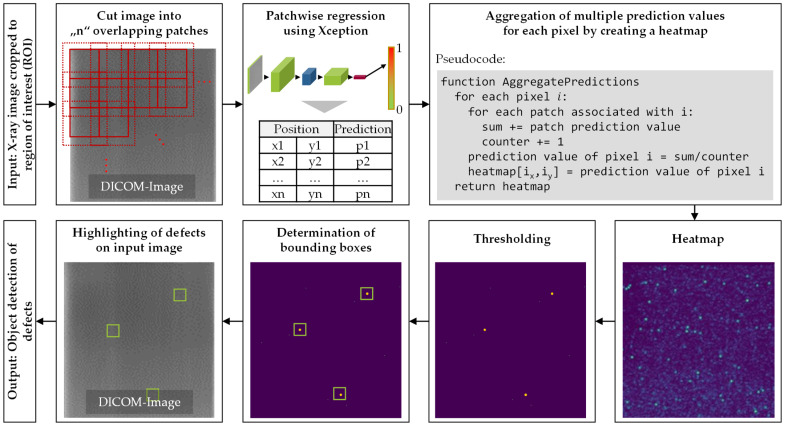
Methodology of the object detection algorithm.

The developed concept is mainly based on the sliding window approach of [25] which uses a 2D scalar representation for recombining the prediction results. For the patchwise regression, the state-of-the-art Xception algorithm [22] mentioned in Section 2.2 is applied, which has delivered the best results in preliminary tests as described in Section 3.4. The main steps of the proposed object detection algorithm are illustrated in Figure 4.

In the first step, the ROI is extracted from the image with Canny edge detection and Hough line transform. The ROI is then cut into overlapping patches using a sliding window approach. These patches serve as input for a regression algorithm, which assigns a value between “0” and “1” to each patch, depending on the severity of defects contained. Due to the overlap, multiple values are determined for each pixel. To recombine these values in the 2D scalar field, the values are normalized for patch number. Combining the results for overlapping patches improves the results for small quantities of training data as it decreases the dependence on the position of the defect within the patch. To distinguish between critical and noncritical defects, a threshold is applied. Based on the resulting binary image, bounding boxes are determined and added to the input image to highlight the detected defects.

### 3.2. Dataset

The available dataset was recorded over a six-year span. All images were recorded with the same X-ray machine on identical settings with the exception of the focus length adjusted to the grid-type-specific length. Two different grid types are analyzed in the proposed application, from here on referred to as grid type 1 and grid type 2.

**Figure 5:**
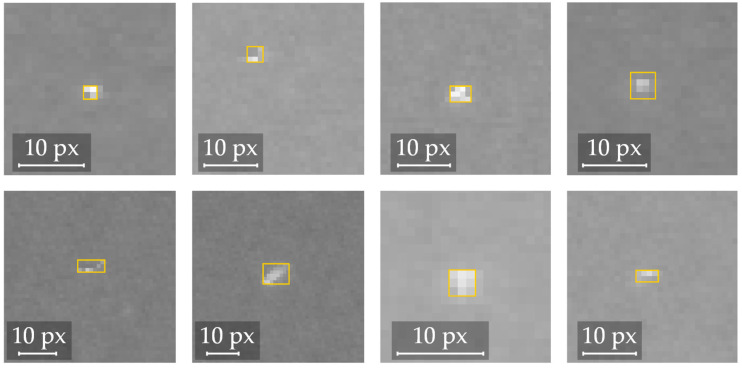
Screenshots of various samples of local defects marked with a bounding box in the labeling tool.

The image data are stored in the DICOM format, an open standard for X-ray images. The images have a bit depth of 12, meaning the values for each pixel range from 0 to 4095. A labeled dataset was created by assessing a total of 140 images (70 of each grid type) and labeling areas with critical local defects with bounding boxes. To ensure consistency, all images were labeled by the same person trained by the regular inspectors. The ground truth was determined by assessing the images on a medical-grade black and white monitor with 2048 × 1536 pixels and 16-bit architecture. The images were displayed in the software tool “eFilm”, and the labeling process was done using “Alp’s Labeling Tool” [30] in Fiji, a version of ImageJ [31]. A few samples of labeled defects are shown in Figure 5. Local defects can vary in size, shape, and distinctness from the noisy surrounding area. In total, the dataset contains over 500 defects.

### 3.3. Training Strategy

The available dataset contains 140 images and their label data of which 120 were used to generate a balanced training dataset of which 75% was used for training and the remaining 25% was used for validation. The remaining 20 images were used as a test dataset. In the preprocessing step, the active area of the grid is extracted from the image, as described in Section 3.1. In the next step, the patches for areas with and without defects for the training and validation datasets were generated. Therefore, the brightest pixel in each bounding box was identified and a random offset within the range of the patch size was applied in both dimensions. The patches without defects are random sections of the preprocessed image, which do not overlap with any bounding box. Five patches containing a defect were generated per bounding box, resulting in 2325 patches with defects. To create a balanced dataset, the same number of patches without defects was uniformly generated from the images. To make the model more robust against stochastic data alterations, the following data augmentation operations were applied: rotation by 90° angles, flipping, and brightness variation. The model was then trained based on the augmented training dataset.

In contrast to the training and validation dataset, the images of the test dataset were cut entirely into patches with an offset of 25%, and predictions were made during inference with the trained model. Since the patches were overlapping, postprocessing as illustrated in Section 3.1 was required. The final evaluation of the model’s performance was then assessed by comparing the final outcome with the labels of the test data.

**Figure 6:**
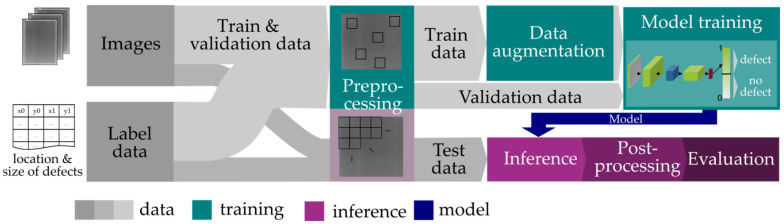
Training strategy.

The used training strategy consists of multiple sequential steps (see Figure 6).

### 3.4. Model Selection and Hyperparameter Optimization

In the model selection process, the custom model architecture and the state-of-the-art image classification model Xception were considered. The custom model consists of seven layers (four convolutional layers, two max-pooling layers, and one fully connected layer). Of the Xception model, two variations were tested. The first is the full Xception model as introduced in [22], and the second is a simplified architecture without the middle flow and without the fully connected layers. The Xception models showed significantly faster convergence and better overall accuracy values than the custom model and were therefore chosen for the following hyperparameter optimization. Data- and model-related parameters considered in the hyperparameter optimization process are summarized in Table 1.

During training, the performance of the two-class classification (defect, no defect) was evaluated using the accuracy value as a training metric. Based on preliminary investigations of the convergence behavior, the maximum amount of training epochs was set to 20.

## 4. Evaluation

The evaluation section is divided into two parts. First, the model performance is evaluated after training as well as after the additional postprocessing step. Secondly, the developed prototype of a visual assistance system is assessed in operation to examine the actual effectiveness for the use case.

### 4.1. Model Evaluation

**Figure 7:**
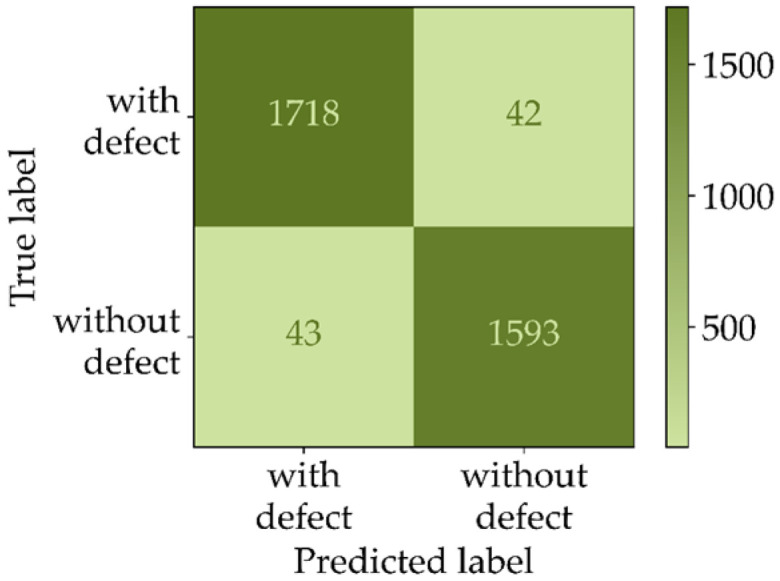
Confusion matrix of the patchwise prediction results of the balanced validation dataset.

The model evaluation after training and after postprocessing was conducted in two different ways. After training, the model performance was determined by evaluating the patchwise prediction results of a balanced validation dataset. Accuracy was chosen as an evaluation metric. Figure 7 illustrates the corresponding confusion matrix of the balanced validation dataset.

**Figure 8:**
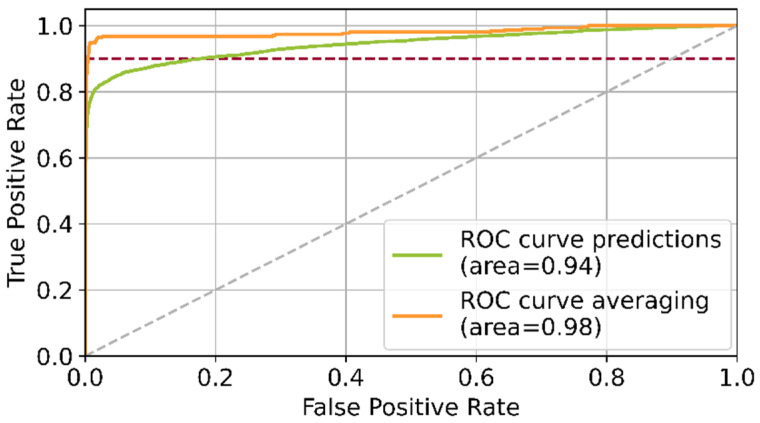
Comparison of the ROC curves of both grid types for patchwise predictions without postprocessing (green) and with postprocessing (orange); applied threshold at recall of 90% indicated in red.

As illustrated by the calculated metrics in Figure 7, the best model found during the parameter optimization achieves an accuracy of 97.6%. When applying the model to entire test images, which have a natural imbalance, the false positive rate raises significantly. To counteract this effect, the postprocessing step described in Section 3.1 was applied. The evaluation results achieved on the imbalanced test dataset with and without postprocessing are illustrated in Figure 8.

Applying the postprocessing leads to a shift of the ROC curve, reducing the false positive rate from 18% to 1% at a fixed target recall of 90%. It can be concluded that the postprocessing step significantly enhances the patchwise prediction results.

**Figure 9:**
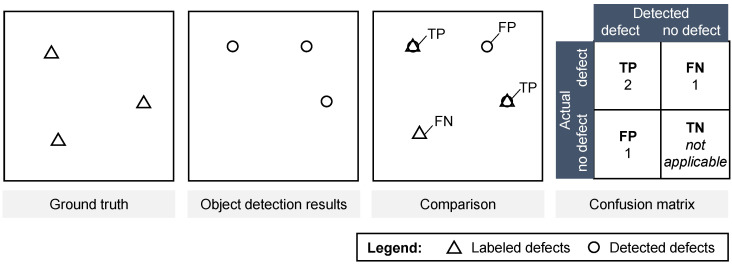
Evaluation procedure of the final examination.

For the final examination of the object detection algorithm, the labeled defects of 20 entirely labeled test images were compared with the defects detected by the algorithm. This procedure is illustrated in Figure 9.

**Figure 10:**
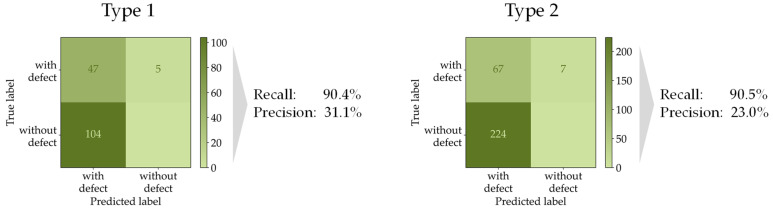
Results of final examination of the object detection algorithm comparing the detected defects with the labeled defects on 10 images per grid type.

As Figure 9 illustrates, the outcome of the evaluation procedure is a confusion matrix, containing the TP, FN and FP counts for the compared detected and non-detected defects. From these values, the sensitivity (recall) and precision metric are calculated as final evaluation metrics. The evaluation procedure was conducted for two different grid types, showing similar results, as visualized in Figure 10.

When adhering to a recall of 90%, a comparatively low precision score of 23% and 31% is achieved. In other words, while detecting 90% of the defects correctly, only one out of four detected defects is actually labeled in the ground truth data. While this amount of false positives is tolerable in the underlying use case, further examinations have shown that shifting the applied threshold in favor of an even higher recall would lead to a disproportionately large increase in false positives.

### 4.2. Evaluation of Prototype

**Figure 11:**
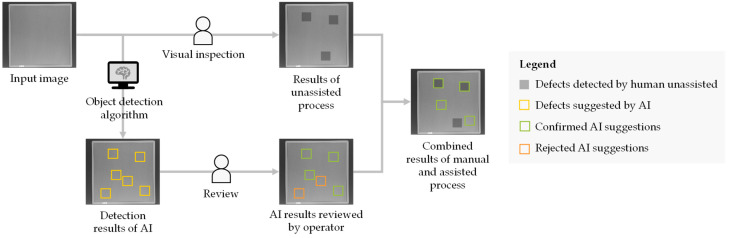
Methodology of the effectiveness examination of the assistance system.

To evaluate the effectiveness of the developed assistance system, seven previously trained testers assessed ten images with and without the assistance of the AI algorithm. As the application serves as an additional support system, the effectiveness of the application was measured by comparing the results of the unassisted process with the combined results of both processes, as illustrated in Figure 11.

**Figure 12:**
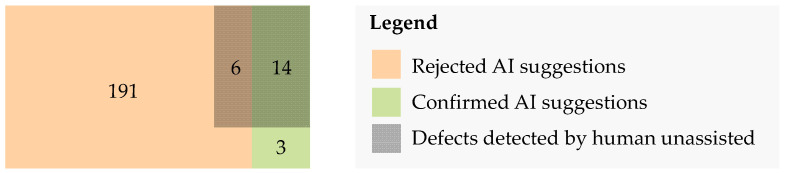
Results of the practical examination; evaluation based on majority vote per classified defect.

In the unassisted process, a tester examines the input images for critical defects. In the assisted process, the operator only reviews the highlighted potential defects suggested by the AI and either confirms or rejects these prediction results. The confirmed defects are then merged with the detected defects of the unassisted process. The improvement achieved through this additional procedure is illustrated in Figure 12.

As Figure 12 summarizes, in total 20 critical defects were identified by the operators in the unassisted process. The AI-based algorithm suggested a total of 214 areas with potential defects of which 197 were rejected by the operators. Six of the rejected suggestions were classified as critical defects in the unassisted process, which may be due to decision fatigue caused by the high number of object areas to be reviewed. Seventeen of the suggested defects were confirmed, three of which were not identified in the unassisted process. All defects detected in the unassisted process were identified by the AI-based algorithm. This corresponds to a recall of 100%. Using the results from the assisted process in a combined manner with the results of the unassisted process, the total amount of critical defects detected increased from 20 to 23, an improvement by 13.4%.

## 5. Conclusions

In this paper, the development and evaluation of an object detection algorithm for X-ray scatter grids are presented. A special characteristic of the developed approach is the applied postprocessing procedure which mitigates the false positive rate, which is due to the extremely high imbalance of the two classes (defect, no defect) during application. The final system is able to detect 90% of the defects at a precision score of approximately 25% for two different grid types. The approach presented in this paper sets a first benchmark for the assisted inspection of X-ray scatter grids.

A practical examination of the effectiveness elaborates the potential of the approach, improving the detection results by over 13%. This improvement comes at the cost of additional efforts for examining all suggestions made by the AI.

Future work will integrate the AI algorithm in a viewer software serving as an interface for the inspection process and a collector of the testers’ review of the AI suggestions. This allows incorporating the testers’ review results into a continuous training cycle further improving the model’s performance.

## Figures and Tables

**Table 1:** Overview of tuned hyperparameters and tested values; chosen values displayed in bold.

Category	Hyperparameter	Considered Values
Data-related	Patch size	16, **32**, 64, 128
	Patches per labeled defect	1, **5**, 15
	Augmentation	**Yes**, No
Model-related	Architecture adjustments	Full Xception, **simplified Xception**
	Regularization function	Without, L1 norm, **L2 norm**

## Data Availability

Data are entirely commercial property and will be not available.
